# Multistrain genome analysis identifies candidate vaccine antigens of *Anaplasma marginale*

**DOI:** 10.1016/j.vaccine.2011.04.131

**Published:** 2011-07-12

**Authors:** Michael J. Dark, Basima Al-Khedery, Anthony F. Barbet

**Affiliations:** a Department of Infectious Diseases and Pathology, College of Veterinary Medicine, University of Florida, USA; b Emerging Pathogens Institute, University of Florida, USA

**Keywords:** omp, outer membrane protein, msp, major surface protein, SNP, single nucleotide polymorphism, *Anaplasma marginale*, Genomics, Rickettsiae, Vaccine candidates, Bacteria

## Abstract

Anaplasmosis in domestic livestock is an impediment to animal health and production worldwide, especially in developing countries in Africa, Asia, and South America. Vaccines have been developed and marketed against the causative organism, *Anaplasma marginale*; however, these have not been widely used because of breakthrough infections caused by heterologous strains and because of the risk of disease induced by live vaccine strains themselves. Recently, molecular studies have enabled progress to be made in understanding the causes for breakthrough infections and in defining new vaccine targets. *A. marginale* has a system for antigenic variation of the MSP2 and MSP3 outer membrane proteins which are members of the *pfam01617* gene superfamily. In this study, we used high throughput genome sequencing to define conservation of different superfamily members in ten U.S. strains of *A. marginale* and also in the related live vaccine strain *A. marginale* subspecies *centrale*. The comparisons included the pseudogenes that contribute to antigenic variation and other superfamily-encoded outer membrane proteins. Additionally, we examined conservation of other proteins proposed previously as vaccine candidates. These data showed significantly increased numbers of SNPs in *A. marginale* subspecies *centrale* when compared to all U.S. *A. marginale* strains. We defined a catalog of 19 conserved candidate vaccine antigens that may be suitable for development of a multi-component recombinant vaccine. The methods described are rapid and may be suitable for other prokaryotes where repeats comprise a substantial portion of their genomes.

## 1 Introduction

*Anaplasma marginale* is a pathogen of cattle in the Order *Rickettsiales*, causing cyclic anemia and occasionally death. The organism causes severe economic losses in livestock production worldwide [1]. Various strategies have been implemented to develop a vaccine to mitigate the impact of this disease. The first attempt at a vaccine was in the early 1900s, with the isolation of *A. marginale* subspecies *centrale*, a less virulent strain that gives some cross-protection to fully virulent strains [2]. Other vaccine attempts have included a variety of subunit vaccines, none of which provided complete protection against heterologous challenge [3,4]. In addition, while infection with one strain of *A. marginale sensu stricto* typically precludes infection with another, multiple cases of superinfection have been described [5–7].

Vaccine failures are due to expression of variants of the major surface proteins MSP2 and MSP3. *A. marginale* creates a wide array of antigenic variants by substitution of whole or partial pseudogene cassettes into a single genomic expression site by segmental gene conversion [8–11], with increasing complexity of the expressed mosaic proteins [12]. Following persistent infection, the immune system has been exposed to a majority of the simple variants, which prevents another strain with similar variants from establishing concurrent infection. However, if the second strain has a unique pseudogene, novel variants generated by segmental gene conversion allow superinfection to take place [13].

In addition to MSP2 and MSP3, a variety of other variable surface antigens have been found in *A. marginale*; these have been called the *msp2* superfamily [14]. Generally, these are all members of the *pfam01617* (Surface Ag 2), which has related members in several other bacterial genera. Several of these have been found in cross-linked surface antigen complexes, and have been suggested as vaccine candidates [15]. A recent study by Agnes et al. used sera from cattle infected with *A. marginale* subspecies *centrale* to determine antigens that are cross-protective from *sensu stricto* challenge [16]. Several other studies have implicated components of the bacterial type 4 secretion system as vaccine candidates [17–19].

In this paper, we examine multiple strains of *A. marginale sensu stricto*, using high-throughput sequencing techniques to examine the members of the *pfam01617* family and the other previously suggested vaccine components to determine their degree of conservation. Proteins that are widely conserved between all strains are candidates for inclusion in cross-protective vaccines. Further, the techniques described can be used to examine other organisms with significant numbers of repeats, allowing rapid determination of conserved proteins for diagnosis and vaccine development.

## 2 Materials and methods

### 2.1 DNA isolation and genome sequencing

*A. marginale* genomic DNA was isolated from highly infected bovine blood taken at the acute stage of infection. Organisms were purified from uninfected erythrocytes and white cells by passage through a cellulose column (C-6288, Sigma, St. Louis, MO) and frozen [20]. Genomic DNA was isolated from organisms using Qiagen genomic DNA kits according to manufacturer protocols. To compare genomes of Florida and Florida-relapse strains bovine #205 was infected with the Florida strain and experienced maximum acute stage parasitemia of 4% on day 37 post-infection and a minimum packed cell volume (pcv) of 18.5% which resolved to the carrier state, with pcv values returning to 35% and no microscopically detectable parasitemia. Bovine #205 was kept in isolation and splenectomized on day 104 post-infection to allow disease recrudescence. Infected blood from the Florida-relapse strain was obtained on day 129 post-infection at 22.5% parasitemia and 23% pcv. *A. marginale* strains analyzed in the present study were Puerto Rico, Mississippi, Virginia, Florida, Florida-relapse, Florida-Okeechobee, St. Maries-Idaho, South Idaho, Oklahoma and Washington-O. Isolated DNA was provided to the Interdisciplinary Center for Biotechnology Research (ICBR) core facilities, University of Florida for library construction and sequencing on the Roche/454 Genome Sequencer according to standard manufacturer protocols. The SFF format flow files were returned by ICBR for bioinformatics analysis.

### 2.2 Bioinformatics

MosaikAligner was used to align individual reads with the reference genome sequences [21]. The SFF flow files were first combined and converted to .fasta and .qual files using Roche/454 Genome Sequencer FLX System software, version 2.3. MosaikBuild (http://code.google.com/p/mosaik-aligner/) was used to convert reads and the reference sequences to the Mosaik binary format (.dat files). The alignment parameters were: hash size (−hs), 11; maximum percentage of the read length allowed to be errors (−mmp), 0.05; alignment candidate threshold (−act), 20; alignment mode (−m), all. The reference genomes were *A. marginale* St. Maries, Idaho strain, GenBank CP000030; *A. marginale* Florida strain, CP001079 and *A. marginale* subspecies *centrale* Israel strain, CP001759. MosaikText was used to convert the aligned binary data file to the text-based BAM format (−bam) and samtools [22] to sort and index the BAM file for viewing in Artemis [23,24]. Artemis allows viewing of the alignment of individual reads either zoomed in to detect gaps in alignment with respect to the annotated reference sequence or zoomed out to show SNPs over large genome regions. For these analyses, two corrections were made to the GenBank annotations:

1. An *msp3* pseudogene is not annotated in CP001079, complement #46310–47887. This was annotated here as AMF_1097;
2. In CP000030, an *msp3* pseudogene, AM1345, is incorrectly annotated as #1181002–1182983. This was corrected to complement #1181022–1183055, retaining the designation of AM1345.

To define the sensitivity for detecting variant genes by Mosaik alignments, we extracted all variable regions for *msp2* and *msp3* pseudogenes from the three fully sequenced genomes and compared their sequence identities. This was done in an all-against-all analysis of the 22 total *msp2* pseudogenes and 22 total *msp3* pseudogenes in the three sequenced genomes using a MATGAT matrix [25]. From this analysis we determined that the closest matches for variable regions of *msp2* pseudogenes in heterologous genomes ranged from 100 to 73% identity and was 100 to 52% identity for *msp3* pseudogenes (see Table 1). We defined the variable regions as the sequence encoding LGKELAY to MAGNIN for *msp2* pseudogenes and that encoding LETEEL to KNRG for *msp3* pseudogenes. These sequences vary slightly between pseudogenes, for example is more typically LQAEEI to KNRG for *msp3* pseudogenes from *A. marginale* subspecies *centrale*, but the locations can readily be identified by alignment. Comparing pyrosequencing data to all the known *msp2* and *msp3* genes showed that all *msp2* pseudogenes with the best match in the heterologous strain below 92% variable region identity were detected as absent (−) and all *msp3* pseudogenes with below 97% variable region identity were detected as absent (−) (Table 1). Since the Mosaik alignment parameter −mmp allows a 5% error in aligning reads, we conservatively estimate that variant genes are detected as absent if they have <90% identity, but may not be detected as absent if they have >90% identity. In this study we examined the presence or absence of the pfam01617 superfamily including genes encoding OMPs 1 through 15, OPAG1-3 and MSP4 [14,26]; proteins identified by surface cross-linking including their encoding genes AM366, 712, 779, 780, 854, 1011, 1051 [15]; and type 4 secretion system genes AM030, 097, 810, 811, 812, 813, 814, 815, 1053, 1312, 1313, 1314, 1315, 1316 [19]. Numbering refers to annotations of the St. Maries, Idaho strain, CP000030. To be defined as conserved in *A. marginale* in Table 4 no segment of the genes was detected as absent in any comparisons of pyrosequenced data from each of 10 U.S. strains of *A. marginale* with the fully sequenced genomes of Florida and St. Maries, Idaho strains. Pyrosequencing data was previously obtained for *A. marginale* strains Puerto Rico, Mississippi and Virginia and in the present study for *A. marginale* strains Florida, Florida-relapse, Florida-Okeechobee, St. Maries-Idaho, South Idaho, Oklahoma and Washington-O. The average genome coverages were 40×, 12×, 63×, 59×, 76×, 47×, 117×, 37×, 96×, and 108× for the ten strains, respectively, when compared to the completed genome from the Florida strain. Since we did not have current access to the Mississippi strain and coverage was lower for this strain, we also verified that no gene was determined as not conserved solely because of absence in this one strain.

The number of high confidence differences between strains (Table 3) was analyzed using Roche/454 gsMapper software to generate the 454HCDiffs.txt file. The base differences and their locations were extracted with the unix grep command and imported into Excel 2008 (Microsoft, Redmond, WA). The number of differences and their respective frequencies (the percentage of different reads versus total reads that fully span the difference location) were tabulated.

Finally, for coverage and SNP analyses in Fig. 4 and Table 5, the BAM files generated by Mosaik were processed by samtools version 0.12 to generate pileups. Pileups for genes of interest were extracted to determine coverage for each nucleotide position comparing to both the Florida and St. Maries strains. Final coverages for each gene of interest were graphed using Excel 2008. For SNP analysis, raw SFF files were processed by Genomics Workbench (CLC Bio, Aarhus, Denmark), and the output of the SNP identification pipeline was placed into a MySQL database. To increase the stringency of SNP identification, the database was queried for SNPs identified by samtools, and only SNPs identified by both methods are included in the final analysis.

## 3 Results

### 3.1 Comparison of pyrosequencing with Sanger sequencing

Two complete genome sequences of *A. marginale* strains from the United States (Florida and St. Maries, Idaho) and one of *A. marginale* subspecies *centrale* (Israel) are available [14,26,27]. We analyzed high-throughput sequencing data from the Roche/454 instrument on 10 U.S. *A. marginale* strains, including the previously genome-sequenced Florida and St. Maries strains as controls. Including Florida and St. Maries strains enables a comparison to be made between the new pyrosequencing data and data obtained using Sanger sequencing. We included in this comparison a second Florida strain (Okeechobee) and a second Idaho strain (South Idaho). We also included a Florida relapse strain derived from a persistently infected animal after 129 days of infection, to examine genome changes over a short time period.

**Fig. 1:**
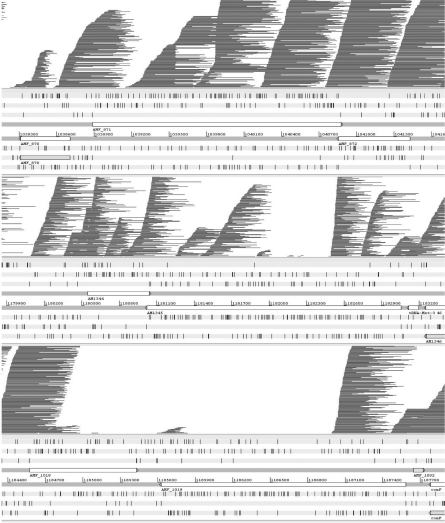
Diversity in *msp2/msp3* gene pairs between *A. marginale* strains. Top panel, pyrosequences of Florida strain compared with the *msp2/msp3* gene pair AMF_871/872 of the Florida strain genome in Artemis. Both genes are defined as present. Middle panel, pyrosequences of Florida strain compared with *msp2/msp3* gene pair AM1344/1345 of the St. Maries, Idaho strain genome. *Msp2* is defined as present, *msp3* as absent. Bottom panel, pyrosequences of St. Maries, Idaho strain compared with *msp2/msp3* gene pair AMF_1018/1019 of the Florida strain genome. Both genes are defined as absent.

The initial analyses compared the original genome sequences with the new pyrosequencing data. This was done by aligning individual pyrosequenced reads with the completed genomes using Mosaik, with visualization of the finished alignments using Artemis. To deal with the known problem of multiple repeats in these genomes, the alignment parameters were set to allow reads to align at multiple different positions in the genome, if this was necessary. A typical result showing alignments with *msp2* and *msp3* genes is shown in Fig. 1. The top panel shows alignment of Florida strain pyrosequencing data with a region of the Florida genome containing an *msp2/msp3* gene pair (AMF_871/872). The reads align over the complete *msp2* and *msp3* regions, as expected. In the middle panel, a comparison is made between the same Florida strain pyrosequencing data but with a region of the St. Maries, Idaho strain genome encompassing the *msp2*/*msp*3 gene pair AM1344/1345. In this case, the previously obtained genome data shows that AM1344 has an exact match (100% identity) with an *msp2* copy in the Florida strain genome, but the closest match of the St. Maries *msp3* copy AM1345 is to an *msp3* copy in the Florida strain with only 78% identity (Table 1). This is revealed by a gap in the aligning sequence reads over the central (hypervariable) region of AM1345, but no gap over AM1344. The lowest panel shows an extreme case where neither the *msp2* (AMF_1018) nor the *msp3* (AMF_1019) pseudogene from the Florida strain aligns with reads from St. Maries. Comparison of the two genome sequences reveals closest matches between the two genomes of 91% for AMF_1018 and 55% for AMF_1019. This analysis was conducted for all *msp2* and *msp3* copies in the three genomes, *A. marginale* (Florida strain), *A. marginale* (St. Maries, Idaho strain) and *A. marginale* subspecies *centrale* (Israel strain). The data revealed that all *msp2* and *msp3* differences with <90% identity were accurately detected (Table 1).

### 3.2 Genome diversity of Anaplasma sp.

We then compared the *msp2* and *msp3* pseudogenes in all 10 U.S. strains of *A. marginale* and *A. marginale* subspecies *centrale*, by the same method (Table 2). The results showed that no *msp2* or *msp3* pseudogene from any of these strains of *A. marginale* from the United States was shared with *A. marginale* subspecies *centrale*. Indeed, there was substantial variation in the repertoire of the *msp2* and *msp3* pseudogenes even within U.S. *A. marginale* strains, with no *msp2* or *msp3* copy shared between Oklahoma and St. Maries, Idaho strains and only one of each shared between Oklahoma and Florida strains. Interestingly, there was substantial variation even between strains from the same state, with no *msp3* pseudogene shared between the two strains from Idaho and only two *msp3* pseudogenes shared between the two strains from Florida (Okeechobee and Florida). In contrast, there was no variation detected between Florida and Florida relapse strains, suggesting that the differences observed reflected evolutionary changes rather than, for example, continuous variation by gene conversion among pseudogenes. It is known from previous analyses that *msp2* and *msp3* expression site sequences are different in Florida and Florida-relapse strains [10,11]. The most conserved *msp2* or *msp3* pseudogene was AM1250, absent in only 2/10 strains examined (WA-O and OK).

We examined whether the diversity observed in *msp2* and *msp3* genes was also reflected in differences in SNP profiles across the genome. High confidence differences between the genomes obtained using Roche/454 gsMapper software are shown in Table 3. Again, few differences were detected between the previous Sanger and current Roche/454 data. Only 38 differences (at 100% frequency) were detected in the Florida strain genome and 84 in the St. Maries, Idaho genome by the two sequencing strategies. Similarly, there were few differences in the Florida relapse strain compared to Florida. Therefore, pyrosequencing data correlated well with the previously reported sequences from traditional Sanger sequencing. Comparison of pyrosequencing of the Florida strain with the previously reported sequence (CP001079) shows high confidence differences, possibly due to true SNPs or error, of one base per 31,643 nucleotides (at 100% frequency), while comparison of pyrosequencing of the St. Maries strain with the previously reported genome sequence (CP000030) yields a difference of one base per 14,258 nucleotides (at 100% frequency). As seen in previous strain comparisons [27], the number of single nucleotide polymorphisms (SNPs) between U.S. strains of *A. marginale* is variable, from 0.20% to 0.58% of the genome. However, all strains of *A. marginale sensu stricto* have significantly increased numbers of SNPs when compared to the *A. marginale* subsp. *centrale* strain, ranging from 1.8% to 2.2%.

**Fig. 2:**
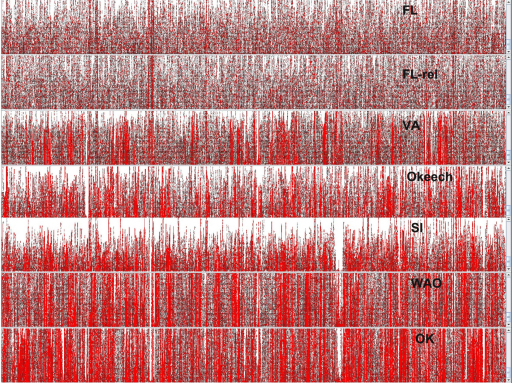
Mosaik alignment of pyrosequencing reads with the fully Sanger-sequenced Florida strain genome to show SNPs. The approximately one third of the genome preceding the origin of replication in the Florida strain is shown on the *x*-axis and the individual pyrosequencing reads for each comparison strain on the *y*-axis. SNPs are shown in red using Artemis. SNPs are distributed throughout the genomes.

**Fig. 3:**
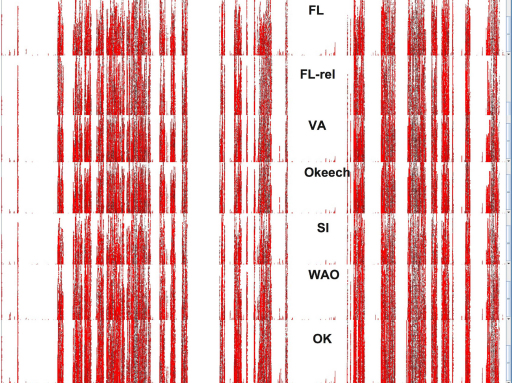
A similar comparison to Fig. 2, but of pyrosequencing reads from multiple *A. marginale* strains with the fully Sanger-sequenced *A. marginale* subspecies *centrale* strain. In addition to SNPs throughout the genomes, there are many gaps in all *A. marginale* strains where no pyrosequencing reads aligning to *centrale* are found.

To visualize the location of differences at the entire genome level, we utilized the “show SNP marks” feature of Artemis for visualizing BAM alignments (Fig. 2). The figure shows the 1/3 of each genome immediately preceding the origin of replication, with SNPs in red. The data show that SNPs are distributed across the genome and agree with Table 3. For example, pyrosequencing data for Florida and Florida-relapse strains closely resemble the genome data derived by Sanger-based sequencing. Furthermore, comparison of Fig. 2 with Tables 2 and 3 clearly reveals the more closely related strains to Florida, i.e. Florida-relapse and Virginia and the more distantly related strains Oklahoma, Washington-O and South Idaho. These relationships are also seen in both SNP numbers and in shared *msp2* and *msp3* pseudogenes. A similar SNP comparison of U.S. strains of *A. marginale* with *A. marginale* subspecies *centrale* (Fig. 3) shows widely distributed SNPs and many gaps between *marginale* and *centrale* where there are no aligning reads. The locations of these gaps were largely identical for all the U.S. *A. marginale* strains, indicating a more distant sequence relationship between all these strains and the *A. marginale* subspecies *centrale* strain.

### 3.3 Conservation of genes encoding proposed vaccine antigens

**Fig. 4:**
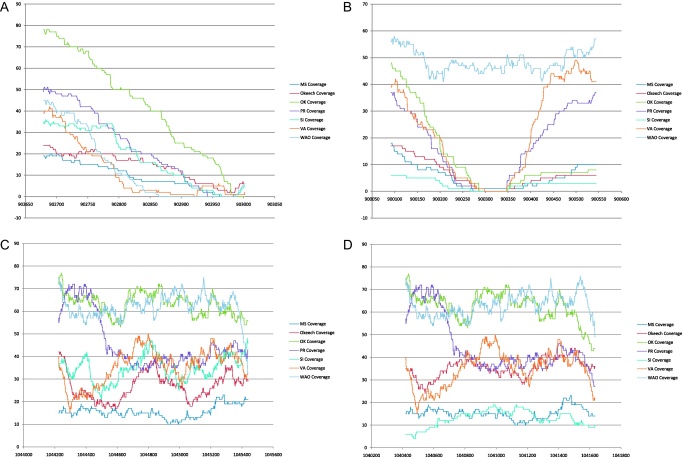
Genome coverage of pyrosequencing reads over *omp15* (A – Florida, B – St. Maries) and *omp4* (C – Florida, D – St. Maries) genes from Florida and St. Maries, Idaho strains of *A. marginale*. Genome position is shown on the *x*-axis and coverage on the *y*-axis. There are no reads aligning with *omp15* in many strains over either the 3′ or central region of the gene, while *omp4* has matching reads throughout its length.

We next examined the conservation of proposed vaccine antigens from the *pfam01617* family, or that have been identified by other strategies. These other strategies involved cross-linking of surface proteins on live organisms by bifunctional reagents, analysis of T-cell responses of immunized and protected animals and identification of components of the type 4 secretion system recognized by T cells [14,15,17–19,26]. The data identified several proteins in each class that were conserved among all 10 U.S. strains of *A. marginale* (Table 4). Interestingly, none were conserved with *A. marginale* subspecies *centrale*. This suggests that relying only on antigens shared between *marginale* and *centrale* may not be an optimal strategy for development of vaccines against U.S. strains of *A. marginale*. Additionally, comparison of the newly sequenced strains with the previously sequenced strains showed multiple genes that are variable in one or more strains; however, no candidate antigen gene was defined as absent in all the newly sequenced strains. Some genes, such as *omps2, 7, 8* and *15* were more frequently detected as absent, whereas others, such as *omps10* and *14*, were detected as absent in only three comparisons between different *A. marginale* strains. An example of detailed coverage graphs for *omp4* (conserved in all strains) and *omp15* (variable) genes is shown in Fig. 4. Although *omp15* coverage graphs suggest variability of this gene in most strains, the variability is localized to the C-terminus when all strains are compared to Florida and to the central region of *omp15* when compared to St. Maries. It should also be recognized that despite their overall conservation and definition as present, non-synonymous SNPs are present in most of the candidate antigen genes (Table 5). There appears to be no trend towards increased numbers of SNPs or decreased conservation when comparing *omps* that are transcribed in either ticks or cattle [33].

## 4 Discussion

Development of vaccines against anaplasmosis has received considerable attention over the last 50 years and has resulted in several marketed live and inactivated whole-organism vaccines [28]. None are currently available in the U.S. because of varying efficacy against heterologous strains and/or side-effects such as isoerythrolysis due to contaminating erythrocyte proteins in the vaccines. This has stimulated the search for improved vaccines and also attempts to understand the reasons for the breaks in vaccine protection against heterologous strains [29–31].

The reason for breaks in protection appear to be due to a sophisticated system for antigenic variation, whereby the expressed MSP2 and MSP3 outer membrane proteins continually change in sequence [32]. This is caused by segmental gene conversion of genomic expression sites for MSP2 and MSP3 by genomic pseudogenes [10]. The repertoire of pseudogenes determines the ability of an incoming strain to superinfect a persistently infected carrier animal [13]. We show here that the pseudogene repertoire is extremely diverse for both MSP2 and MSP3 across the U.S., even within *A. marginale* strains from the same state. No *msp2* or *msp3* pseudogene was present in all U.S. strains. Therefore, it is unlikely that a vaccine could be developed by trying to include a full repertoire of potential MSP2/MSP3 variants in a vaccine. However, other members of *pfam01617* (to which both *msp2* and *msp3* belong) encode conserved OMPs and are expressed in *A. marginale* [33] and, therefore, still remain viable vaccine candidates.

Two other vaccine strategies have also been proposed recently. The first [16] relies on the protection afforded by the less virulent strain *A. marginale* subspecies *centrale*. This strain has been extensively used in the field in Australia, South Africa, Argentina, Uruguay, Israel, Zimbabwe and Malawi. Recent research has found proteins with immunogenic epitopes shared between *marginale* and *centrale*, although the overall protein sequence identities were less than 90% [16], and these have been proposed for inclusion in a subunit vaccine. Although *A. marginale* subsp. *centrale* undoubtedly provides some protection against *A. marginale* strains [35], controlled trials have shown low efficacy of this vaccine against heterologous isolates from South America and Africa [36–39], and infection by *A. marginale* subspecies *centrale* does not prevent subsequent superinfection by *A. marginale* [40]. These data have stimulated the search for less virulent strains of *A. marginale* to potentially replace the *A. marginale* subspecies *centrale* vaccine, and such strains have been identified in Australia and Mexico [41,42].

The second, recently proposed, vaccine strategy relies on the observation that immunization with inactivated organisms or outer membranes can induce sterile protective immunity against challenge with *A. marginale* [3,43]. Two investigations are particularly noteworthy in this regard: firstly, the identification of the surface proteome of *A. marginale* [15,17] and secondly, the identification of type 4 secretion system components recognized by T and B cells from protected cattle [19]. However, while sterile immunity against homologous challenge has been achieved, these provide only partial immunity against heterologous challenge. This may be due to the immunodominant responses induced against the hypervariable MSP2 and MSP3 proteins. Compared to these, other antigens, such as the T4SS proteins and other surface proteome molecules, are considered subdominant antigens. These induce weaker and more inconsistent antibody and T cell responses, at least in the context of complex immunogens such as whole organism and membrane vaccines that also contain MSP2 and MSP3 [19]. However, while these responses may be less robust, these antigens appear to be less variable, making them important to include in a vaccine producing pan-strain immunity.

The body of previous research in *A. marginale* has resulted in a large catalog of potential vaccine candidates. We attempted here to reduce the number of candidate antigens by applying high throughput genome sequencing and bioinformatics analysis to 10 U.S. strains of *A. marginale*. The intent was to identify the most conserved proteins from all of the above vaccine strategies that may form the core components of a broadly protective vaccine.

We initially verified that pyrosequencing was capable of accurately determining the relationships among already fully sequenced strains and the variable *msp2* and *msp3* pseudogenes in those strains. We correctly identified the shared *msp2* and *msp3* pseudogenes and those having <90% identity. This method was then applied to all 10 U.S. strains of *A. marginale*. Extensive diversity was observed in the repertoire of both *msp2* and *msp3* pseudogenes among strains, with generally more diversity observed in the complement of *msp3* pseudogenes when compared to *msp2*.

There was also extensive diversity in SNPs among strains, distributed over most of the genome, agreeing with previous observations on a smaller subset of strains [27]. However, the members of the *pfam01617* family are relatively well conserved overall, with no protein having <90% identity between all the strains examined. All of these proteins have SNPs, and SNPs within strains have a similar distribution pattern to those described for the rest of the genome in terms of the numbers of strains with polymorphisms.

A surprising observation was the more extensive diversity in *A. marginale* subspecies *centrale* when compared to all 10 U.S. *A. marginale* strains. The taxonomic position of *centrale* compared to *marginale* has been debated previously, with some investigators proposing a separate species, *Anaplasma centrale* [44–46]. However, only a few strains of *A. marginale* subspecies *centrale* are available for analysis. We suggest that resolution of this question should await genomic data on non-U.S. strains of both *marginale* and *centrale*, particularly strains from Africa. This would resolve whether there is a continuum of strain diversity among *marginale* strains eventually reaching that of the single currently sequenced *centrale* strain, originally isolated by Theiler in South Africa. A recent study [47] comparing membrane proteins from a Brazilian strain of *A. marginale* with Florida and St. Maries determined amino acid sequence identities of 92–100% for all OMPs investigated except OMP7, compared to 40–70% identities with the *A. marginale* subspecies *centrale* orthologs. This suggests that the diversity observed here among U.S. strains of *A. marginale* may at least be representative of *marginale* strains in North and South America.

Finally, the data reveal the candidate vaccine antigens conserved among U.S. strains of *A. marginale*. The catalog includes conserved members of *pfam01617*, as well as components of the bacterial type 4 secretion system and proteins identified by surface cross-linking. Interestingly, it does include three proteins identified previously that contain epitopes shared with *A. marginale* subspecies *centrale*, namely OMP11 (AM1255), AM779 and AM854 [16]. However, overall the list is broader than just the antigens conserved between *A. marginale sensu stricto* and subspecies *centrale*. It also eliminates less conserved proteins and housekeeping genes which share epitopes between *centrale* and *marginale*. Additionally, although conserved, OMP6 and OPAG1 can probably be eliminated from consideration as vaccine candidates as no expressed peptides were detected from the encoding genes in any life cycle stages in prior studies [33,34]. This revised catalog of 19 antigens (see Table 4) would be readily approachable for synthesis by recombinant expression technology and inclusion in a multi-component vaccine for testing. The present genomic data and previous experimental data suggest that such a vaccine may be efficacious against U.S. strains of *A. marginale*.

These data also illustrate the utility of next-generation sequencing techniques for identification of antigens and epitopes conserved between multiple strains. While rapid sequencing has been used extensively, this study shows its utility in examination of repetitive genes. While these techniques cannot yet assemble a genome through extensive repetitive regions, they can show regions where there is genetic similarity or where homologous regions are missing in newly sequenced strains.

## Figures and Tables

**Table 1:** Comparison of *msp2* and *msp3* genes between pyrosequenced and Sanger-sequenced *Anaplasma* genomes. Genes detected as present (+) or absent (−) by pyrosequencing are shown together with their % sequence identity (of the best match, by MATGAT), in parentheses. The respective gene annotations are shown at the top. All genes with <90% sequence identity between strains were detected as absent. Mean genome coverage for pyrosequencing data is indicated in the left column. *Genome abbreviations*: FL – Florida, StM – St. Maries, ACIS – *A. marginale* subspecies *centrale*, Israel.

	FL msp2								StM msp2							ACIS msp2						
Pyro																						
Annot.	25	47	155	534	757	872	945	1018	33	49	213	720	1152	1250	1344	29	47	50	145	601	1176	1183
FL	(100)	(100)	(100)	(100)	(100)	(100)	(100)	(100)	(99)	(92)	(78)	(92)	(100)	(100)	(100)	(79)	(76)	(79)	(75)	(81)	(79)	(77)
59X	+	+	+	+	+	+	+	+	+	+	−	+	+	+	+	−	−	−	−	−	−	−
FL-rel																						
76X	+	+	+	+	+	+	+	+	+	+	−	+	+	+	+	−	−	−	−	−	−	−
StM	(99)	(92)	(90)	(92)	(100)	(100)	(100)	(91)	(100)	(100)	(100)	(100)	(100)	(100)	(100)	(78)	(74)	(78)	(73)	(80)	(79)	(77)
117X	+	+	−	+	+	+	+	−	+	+	+	+	+	+	+	−	−	−	−	−	−	−

	FL msp3							StM msp3							ACIS msp3							
Pyro																						
Annot.	34	58	65	533	871	1019	1097	47	62	78	91	718	1150	1345	70	144	298	354	393	1076	1173	1180
FL	(100)	(100)	(100)	(100)	(100)	(100)	(100)	(100)	(60)	(57)	(76)	(100)	(97)	(78)	(58)	(58)	(59)	(54)	(57)	(52)	(60)	(59)
59X	+	+	+	+	+	+	+	+	−	−	−	+	+	−	−	−	−	−	−	−	−	−
FL-rel																						
76X	+	+	+	+	+	+	+	+	−	−	−	+	+	−	−	−	−	−	−	−	−	−
StM	(59)	(56)	(68)	(100)	(97)	(55)	(100)	(100)	(100)	(100)	(100)	(100)	(100)	(100)	(59)	(58)	(59)	(53)	(61)	(52)	(60)	(59)
117X	−	−	−	+	+	−	+	+	+	+	+	+	+	+	−	−	−	−	−	−	−	−

**Table 2:** Shared *msp2* and *msp3* pseudogenes between U.S. *A. marginale* strains and *A. marginale* subspecies *centrale* (Israel strain). No pseudogenes are shared between any of ten U.S. *marginale* strains and *central*e. The repertoire of both *msp2* and *msp3* pseudogenes is diverse in U.S. *marginale* strains. Strain abbreviations: FL – Florida, WA-O – Washington-Okanagan, OK – Oklahoma, VA – Virginia, MS – Mississippi, SI – South Idaho, StM-I – St. Maries, Idaho, ACIS – *A. marginale* subsp. *centrale*, Israel.

	FL msp2	FL msp3	StM-I msp2	StM-I msp3	ACIS msp2	ACIS msp3
FL	8/8	7/7	6/7	3/7	0/7	0/8
FL-relapse	8/8	7/7	6/7	3/7	0/7	0/8
FL-Okeechobee	4/8	2/7	3/7	2/7	0/7	0/8
WA-O	2/8	0/7	2/7	0/7	0/7	0/8
OK	1/8	1/7	0/7	0/7	0/7	0/8
VA	6/8	2/7	4/7	2/7	0/7	0/8
MS	1/8	0/7	2/7	0/7	0/7	0/8
Puerto Rico	8/8	3/7	6/7	2/7	0/7	0/8
SI	2/8	0/7	2/7	0/7	0/7	0/8
StM-I	6/8	3/7	7/7	7/7	0/7	0/8

**Table 3:** Numbers of high confidence differences between *Anaplasma* strain genomes (gsMapper software). Total differences as well as non-polymorphic differences (found in all reads covering the respective regions, 100% frequency) are shown. Excluding homologous comparisons (FL, FL-relapse, St. Maries, Idaho), there are an average of 5302 differences between *A. marginale* strains and 23,984 between *A. marginale* strains and *A. marginale* subspecies *centrale*. Strain abbreviations are as specified in Table 2.

Pyrosequencing data	vs. FL		vs. StM-I		vs. ACIS	
	Total	@100% frequency	Total	@100% frequency	Total	@100% frequency
FL	105	38	7516	5233	29,044	24,014
FL-relapse	122	37	7698	5288	29,541	24,341
FL-Okeechobee	8747	5000	8552	5130	28,967	23,979
WA-O	9632	6240	9112	5847	30,388	23,889
OK	11,333	6635	11,662	6930	29,724	23,267
VA	4932	2368	8094	3884	28,696	23,229
MS	8064	5522	7505	5032	24,004	21,905
Puerto Rico	3217	2816	7164	6367	27,593	26,244
SI	9132	6487	8273	5781	27,955	23,719
StM-I	7747	5577	194	84	29,752	25,254

**Table 4:** Mosaik/Artemis alignment analysis identifies conserved genes encoding candidate vaccine antigens.

	Conserved in *A. marginale*	Identification method	*A. centrale* ortholog	Conserved between *A. marginale/centrale*
*omp1*(AM1139)	Yes	Outer membrane protein	ACIS_00234	No
*omp4*(AM1164)	Yes	Outer membrane protein	ACIS_00227	No
*omp6*(AM1219)^a^	Yes	Outer membrane protein	None	No
*omp11*(AM1255)	Yes	Outer membrane protein	ACIS_00140	No
*omp12*(AM1257)	Yes	Outer membrane protein	ACIS_00139	No
*opag1*(AM1143)^a^	Yes	Outer membrane protein	ACIS_00231	No
*opag2*(AM1142)	Yes	Outer membrane protein	ACIS_00232	No
*opag3*(AM1140)	Yes	Outer membrane protein	ACIS_00233	No
*msp4*(AM090)	Yes	Outer membrane protein	ACIS_01187	No
AM779	Yes	Surface X-linking	ACIS_00557	No
AM780	Yes	Surface X-linking	ACIS_00556	No
*pal*(AM854)	Yes	Surface X-linking	ACIS_00486	No
*purD*(AM1011)	Yes	Surface X-linking	ACIS_00340	No
*virB3*(AM815)	Yes	Type 4 secretion	ACIS_00521	No
*virB4-1*(AM814)	Yes	Type 4 secretion	ACIS_00522	No
*virB4-2*(AM1053)	Yes	Type 4 secretion	ACIS_00304	No
*virB6*(AM811)	Yes	Type 4 secretion	ACIS_00526	No
*virB8-1*(AM1316)	Yes	Type 4 secretion	ACIS_00090	No
*virB9-2*(AM1315)	Yes	Type 4 secretion	ACIS_00091	No
*virB10*(AM1314)	Yes	Type 4 secretion	ACIS_00092	No
*virB11*(AM1313)	Yes	Type 4 secretion	ACIS_00093	No

^a^Not expressed.

**Table 5:** Total and non-synonymous SNPs in the *pfam01617* superfamily and genes encoding candidate vaccine antigens. SNPs – the number of nucleotide positions in each gene with high-confidence changes in at least one other strain (excluding gene segments previously defined as absent). NS – the number of SNPs producing a non-synonymous change in at least one strain. Ave. Freq. – average frequency of reads with each SNP across all strains. Ave Cov. – average coverage at each SNP nucleotide position.

	Florida				St. Maries			
	SNPs	NS changes	Ave. Freq.	Ave. coverage	SNPs	NS changes	Ave. Freq.	Ave. coverage
*omp1*(AM1139)	26	11	99.8	65.8	26	11	99.8	71.9
*omp2*(AM1156)	1	0	100	12	0	0	N/A	N/A
*omp3*(AM1159)	2	2	100	41	1	1	100	70
*omp4*(AM1164)	8	2	99.8	43.5	6	0	99.7	58.9
*omp5*(AM1166)	23	2	99.7	53.9	15	6	99.9	83.2
*omp6*(AM1219)	5	1	100	68	5	1	90.7	104
*omp7*(AM1220)	52	11	99.8	13.3	68	27	96.4	33.9
*omp8*(AM1221)	38	9	93.2	47.2	49	16	96.9	40.4
*omp9*(AM1222)	32	9	98.3	17.2	31	9	97.7	31.4
*omp10*(AM1223)	15	10	99.3	35	15	10	99.7	67.9
*omp11*(AM1255)	26	13	99.8	36	19	10	99.9	30.2
*omp12*(AM1257)	16	5	99.2	31	17	6	99.3	48.9
*omp13*(AM1258)	19	8	99.7	21.1	14	5	99.3	70.2
*omp14*(AM075)	17	7	99.2	75.7	24	12	100	55.1
*omp15*(AM987)	8	5	100	10.3	4	2	99.3	107.7
*opag1*(AM1143)	1	0	100	12	2	2	100	160
*opag2*(AM1142)	4	2	100	77.8	6	4	100	105.2
*opag3*(AM1140)	43	15	99.8	94.3	43	41	99.8	94.3
*msp4*(AM090)	8	0	99.8	58.1	9	1	99.1	42.9
AM779	7	1	100.0	37.1	7	1	100.0	37.1
AM780	8	4	96.9	14.1	31	14	99.2	41.7
*pal*(AM854)	5	3	99.7	60.2	4	2	100.0	58.0
*purD*(AM1011)	29	1	99.4	42.5	30	1	99.5	48.8
*virB3*(AM815)	0	0	N/A	N/A	0	0	N/A	N/A
*virB4-1*(AM814)	8	1	98.5	37.5	4	1	97.2	18.9
*virB4-2*(AM1053)	8	1	98.5	37.5	4	1	97.2	18.9
*virB6*(AM811)	46	26	99.6	36.7	46	25	99.6	39.5
*virB8-1*(AM1316)	2	0	100	35.3	2	0	100	35
*virB9-2*(AM1315)	2	0	97.4	45	2	0	97.4	35
*virB10*(AM1314)	5	2	99.9	57.9	5	2	99.9	51.4
*virB11*(AM1313)	14	1	99.1	52.7	15	1	98.8	46.4
